# Development of End-to-End AI–Based MRI Image Analysis System for Predicting IDH Mutation Status of Patients with Gliomas: Multicentric Validation

**DOI:** 10.1007/s10278-023-00918-6

**Published:** 2024-01-12

**Authors:** João Santinha, Vasileios Katsaros, George Stranjalis, Evangelia Liouta, Christos Boskos, Celso Matos, Catarina Viegas, Nickolas Papanikolaou

**Affiliations:** 1 Computational Clinical Imaging Group, Champalimaud Research , Champalimaud Foundation, Av. Brasília, 1400-038 Lisbon, Portugal; 2 Instituto Superior Técnico, Universidade de Lisboa, Av. Rovisco Pais 1, 1049-001 Lisbon, Portugal; 3 Department of Radiology, General Anti-Cancer and Oncological Hospital of Athens, St. Savvas, Athens, Greece; 4 Department of Neurosurgery, National and Kapodistrian University of Athens, Evangelismos Hospital, Athens, Greece; 5 Hellenic Center for Neurosurgical Research “Prof. Petros Kokkalis”, Athens, Greece; 6 Athens Microneurosurgery Laboratory, Athens, Greece; 7 IATROPOLIS CyberKnife Center, Hellenic Neuro-Oncology Society, Chalandri, Greece; 8 Radiology Department, Champalimaud Clinical Centre, Champalimaud Foundation, Av. Brasília, 1400-038 Lisbon, Portugal; 9 Department of Neurosurgery, Hospital Garcia de Orta, Almada, Portugal

**Keywords:** Radiogenomics, AI, Glioma, IDH mutation status

## Abstract

Radiogenomics has shown potential to predict genomic phenotypes from medical images. The development of models using standard-of-care pre-operative MRI images, as opposed to advanced MRI images, enables a broader reach of such models. In this work, a radiogenomics model for IDH mutation status prediction from standard-of-care MRIs in patients with glioma was developed and validated using multicentric data. A cohort of 142 (wild-type: 32.4%) patients with glioma retrieved from the TCIA/TCGA was used to train a logistic regression model to predict the IDH mutation status. The model was evaluated using retrospective data collected in two distinct hospitals, comprising 36 (wild-type: 63.9%) and 53 (wild-type: 75.5%) patients. Model development utilized ROC analysis. Model discrimination and calibration were used for validation. The model yielded an AUC of 0.741 vs. 0.716 vs. 0.938, a sensitivity of 0.784 vs. 0.739 vs. 0.875, and a specificity of 0.657 vs. 0.692 vs. 1.000 on the training, test cohort 1, and test cohort 2, respectively. The assessment of model fairness suggested an unbiased model for age and sex, and calibration tests showed a *p* < 0.05. These results indicate that the developed model allows the prediction of the IDH mutation status in gliomas using standard-of-care MRI images and does not appear to hold sex and age biases.

**Supplementary Information:**

The online version contains supplementary material available at 10.1007/s10278-023-00918-6.

## Background

Diffuse gliomas in adult patients are the most common primary malignant tumors of the central nervous system (CNS), accounting for more than 70% of primary brain tumors [1]. Diagnosis and management recommendations for patients with diffuse gliomas have been updated, with diagnostic classification undergoing significant changes [2]. The classification, which initially relied solely on morphological and histological characteristics, was revised in 2016 as part of the World Health Organization (WHO) revision of CNS tumor classification, introducing genomic features to improve diagnosis [3]. Later, in 2021, this classification was revised again, resulting in a more prominent use of genomic characteristics in diagnosing patients with glioma. This revision ensures a complete diagnosis by grouping tumors based on genetic modifications (e.g., IDH and H3 status), resulting in only three groups: astrocytoma, IDH-mutant; oligodendroglioma, IDH-mutant and 1p/19q-codeleted; and glioblastoma, IDH-wild-type [4]. As such, the IDH mutation status has become an essential diagnostic criterion, in which IDH-wild-type astrocytomas (WHO grades 2 and 3) with EGFR amplification and/or microvascular proliferation and/or necrosis and/or TERT promoter mutation and/or + 7/ − 10 chromosome copy number changes, are considered to behave as de facto glioblastomas [4, 5].

Radiomics is a promising tool to extract quantitative features from medical images, called radiomic features, which are essential to the patient’s diagnosis, treatment planning, and follow-up. It uses high-throughput analyses, allowing the extraction of many quantitative features, which showed preliminary but promising results in guiding patients’ diagnosis, predicting response to treatment and prognosis, and providing information on cancer genetics [6].

In particular, the study of associations between radiomic features and genomic characteristics or mechanisms, like mutations and methylations, among others, and the consequent development of imaging-based prediction models has been termed radiogenomics [7]. These prediction models are extremely appealing since medical images are an essential part of patient diagnosis, and radiomics provide a noninvasive, less expensive, and less time-consuming assessment of tumors compared to genetic testing. Furthermore, the challenges related to tumoral heterogeneity can be overcome by a non-destructive and 3D assessment of the region of interest, allowing for multiple evaluations during the disease continuum.

Despite the promising results of radiomics, a critical step hindering the adoption of radiomics in a clinical workflow is the region-of-interest segmentation. While manual segmentation is considered the ground truth by many authors, this approach is very labor intensive and shows high intra- and inter-observer variability [8, 9], which has been shown to affect radiomic features and produce significant differences in the performance of classification models [10]. On the other hand, automatic and semi-automatic segmentation methods reduce the annotator interactions improving time efficiency, accuracy, and boundary reproducibility. Additionally, automated segmentation methods have been shown to be indistinguishable from manual segmentation and are currently considered the best option by avoiding intra- and inter-observer variability of radiomic features and subsequently in the developed models [11, 12].

While several studies have investigated the use of deep learning and radiomics to predict IDH mutation status, only a few studies considered all different histological types, according to the WHO CNS 2016/2021 revisions [13–15], and only [13] and [14] evaluated the developed model on independent external test datasets. From these, only [13] used standard-of-care MRI sequences (pre-contrast T1-weighted, fluid-attenuated inversion recovery, T2-weighted, post-contrast T1-weighted) and automatic segmentation, but none investigated the fairness of the developed models.

In this study, an end-to-end AI solution to predict the IDH mutation status from standard-of-care MRI sequences in patients with glioma was developed using a public dataset and was assessed using external multicentric data. This end-to-end AI solution comprises an initial automatic brain extraction, followed by the automatic segmentation of the edema, contrast-enhancing, and non-contrast-enhancing or necrotic tumor parts, avoiding the development of annotator-dependent models and significantly improving the time efficiency of the radiomic process. The radiomic features are extracted from each image sequence and each of these regions and then used to predict the IDH mutation status. Additionally, we assessed the model’s performance in terms of fairness concerning age and sex biases and adherence to the development and validation of the model to the Transparent Reporting of a multivariable prediction model for Individual Prognosis or Diagnosis (TRIPOD) [16].

## Methods

This retrospective study utilized publicly available data as the training set. The developed solution was externally evaluated using retrospective data collected in two other hospitals.

### Study Participants

#### Training Cohort

MRI and genomic data from The Cancer Imaging Archive (TCIA) [17, 18] and The Cancer Genome Archive (TCGA) [19, 20] were searched to select patients with gliomas containing pre-operative MRI images with T1-weighted (T1w), T2-weighted (T2w), fluid-attenuated inversion recovery (FLAIR), and post-contrast T1-weighted (cT1w) sequences, and the IDH mutation status. Acquisition parameters used in each MRI sequence are reported in Table 2 of [21].

Given the WHO CNS 2021 revision, both TCIA/TCGA-GBM and LGG cohorts were considered and analyzed as a single cohort. The final cohort of patients comprised imaging data acquired from four hospitals located in the USA. The number of patients and IDH mutation status distribution per hospital are shown in Table 1.

**Table 1:** Training cohort distribution by hospital and corresponding occurrence of IDH-mutant and wild-type cases per hospital

**Hospital**	**No. of cases**	**No. of IDH-mutant/wild-type**
**Case Western**	20	11/9
**Case Western—St. Joes**	33	29/4
**Henry Ford Hospital**	58	45/13
**Thomas Jefferson University**	31	11/20
**Total**	142	96/46

#### External Test Cohort 1

Data from 59 patients who underwent surgery at Hospital Garcia de Orta, Portugal, between January 1, 2017, and December 31, 2020, were collected. The local IRB approved authorization for the use of data, and informed consent was waived due to the retrospective design. Fourteen patients were considered ineligible due to missing imaging data, while nine were due to missing IDH mutation status. The final test cohort from center 1 included 36 patients (20 men, 16 women, mean age of 54.8 (standard deviation, 16.3) years).

#### External Test Cohort 2

Data from 103 patients who underwent surgery at the General Anti-Cancer and Oncological Hospital of Athens “St. Savvas” were collected between March 1, 2014, and February 28, 2020. The local IRB approved authorization for the use of data, and informed consent was waived due to the retrospective design. Thirty-two patients were considered ineligible due to missing imaging data, while 18 were due to missing IDH mutation status. The final test cohort from center 2 included 53 patients (29 men, 24 women, mean age of 54.0 (SD, 16.8) years).

The acquisition parameters used in each MRI sequence for both external test cohorts are summarized in Supplementary Material Table S2.

Diagnosis using relevant molecular markers was established from tissue samples obtained from upfront maximum tumor resection performed following pre-operative diagnostics using neurological exams and brain MRI. Given the focus on diffuse gliomas in adult patients, only patients older than 18 were considered. The eligibility criteria, radiomic feature extraction parameters, and outcome definition were not changed between the training and external test cohorts.

### Processing Pipeline

All DICOM images were converted from DICOM to NIfTI format using dicom2nifti (https://github.com/icometrix/dicom2nifti) using the reorient option to ensure proper orientation for the proceeding steps. The T1w, T2w, cT1w, and FLAIR images were then skull stripped using HD-BET, which is a freely available deep learning model trained on data from 1568 MRI examinations from 25 institutions [22]. Consequently, the T2w, cT1w, and FLAIR images were registered to the T1w image of each patient. Finally, the four images were input to the HD-GLIO [23] model to segment each patient’s enhancing, non-enhancing/necrotic, and edema tumoral regions. The latter segmentation model was trained using 455 MRI examinations and validated using 2273 MRI examinations from multiple institutions. Segmentations were then transformed into the original space of each image to avoid the influence of several interpolation steps on the radiomic feature values.

#### Radiomic Feature Extraction

Image preprocessing, consisting of image resampling and intensity normalization, and feature extraction were performed using pyradiomics (version 3.0.0—available in a GitHub repository: https://github.com/AIM-Harvard/pyradiomics/releases/tag/v3.0) [24]. Given the anisotropic nature of the images, images had their in-plane resolution downsampled to the lowest in-plane resolution from all images of each sequence, and two-dimensional feature extraction was performed using pyradiomic preprocessing functionalities [25, 26]. Prior to the feature extraction, images also had their intensity normalized using the *z*-score method followed by a scale and shift to ensure that all images had a mean value of 300 and a standard deviation (SD) of 100, ensuring that, under a standard distribution of intensity values, most of them would be between 0 and 600 (mean ± 3 SD—99.73% of the intensities). The segmentations of the enhanced tumor, nonenhanced tumor and necrosis, and edema regions on each of the T1w, cT1w, FLAIR, and T2w images were used to perform the feature extraction. Shape, first-order, gray-level co-occurrence matrix (GLCM), gray-level run length matrix (GLRLM), gray-level size zone matrix (GLSZM), neighboring gray-tone difference matrix (NGTDM), and gray-level dependence matrix (GLDM) features were extracted from the original and filtered images (Laplacian of Gaussian, LoG, with *σ* = {1.015,2,3} mm; wavelet—two levels; local binary patterns—2D and 3D; gradient). For the texture matrices computation, the intensities were discretized using the fixed bin width approach following the results presented in [27, 28]. The values were chosen for each sequence following the pyradiomic documentation. A summary of the feature extraction parameters is shown in Table 2, and the parameter files used for the feature extraction are available in a GitHub repository: https://github.com/JoaoSantinha/End2EndAI_IDH_Prediction.. A total of 18,036 radiomic features were extracted for each patient, comprising 1503 features for each MRI sequence and segmented region. From these, 15 features were of shape category, and, per image type, 18 features were first-order, 24 were GLCM, 16 were GLRLM, 16 were GLSZM, 5 were NGTDM, and 14 features were GLDM.

**Table 2:** List of feature extraction parameters used for each MRI sequence

**Feature extraction parameters**	**cT1w**	**T1w**	**T2w**	**FLAIR**
**Normalization scale**	100	100	100	100
**Voxel array shift**	300	300	300	300
**Resampled pixel spacing (mm)**	1.016 × 1.016	1.016 × 1.016	1.016 × 1.016	1.016 × 1.016
**Resegment range (mode: sigma)**	[− 3, 3]	[− 3, 3]	[− 3, 3]	[− 3, 3]
**Bin width**	5	2	5	5
**LoG sigma (mm)**	[1.016, 2, 3]	[1.016, 2, 3]	[1.016, 2, 3]	[1.016, 2, 3]
**Wavelet number of levels**	2	2	2	2
**Local binary pattern**	[2D, 3D]	[2D, 3D]	[2D, 3D]	[2D, 3D]

### Model Training and Evaluation

The training cohort was used to train a logistic regression classifier using a fivefold stratified cross-validation procedure to optimize the ridge regularization strength to minimize overfitting utilizing the area under the receiver operating characteristic curve (AUC) as the optimization metric. Within the training procedure, features with (near) zero variance (features in which values were the same in at least 95% of the cases) were disregarded, and probability calibration was performed using Platt’s method with the computation of the unbiased predictions from cross-validation, followed by the calibration. Interaction terms were not addressed in the prediction model. To overcome the class imbalance, the values of the IDH mutation status were used to automatically adjust weights inversely proportional to class frequencies in the training data (scikit-learn option class_weight = “balanced”). The final weights of the model were obtained by refitting the logistic regression with the optimal ridge regularization strength to the entire training cohort. The model was trained and evaluated using the scikit-learn library (version 1.1.3) [29]. Given the inexistence of differences in setting, eligibility criteria, predictors, and outcomes, the model was evaluated without any further updates after training. Fairness across gender and age was also assessed using the Fairlearn library (version 0.7.0)[30].

### Statistical Analysis

Patient characteristics were analyzed using standard descriptive statistics. Statistical analysis of continuous variables was performed with the two-sample Welch’s *t*-test, whereas differences in categorical variables were analyzed using a *χ*^2^-test. The Hosmer–Lemeshow test was used to assess the calibration of the model on the external test cohorts. The reported statistical significance levels were all two-sided, set at *α* < 0.05.

The predictive performance of the model was quantified through the AUC of the ROC, accuracy, sensitivity, and specificity.

## Results

**Fig. 1:**
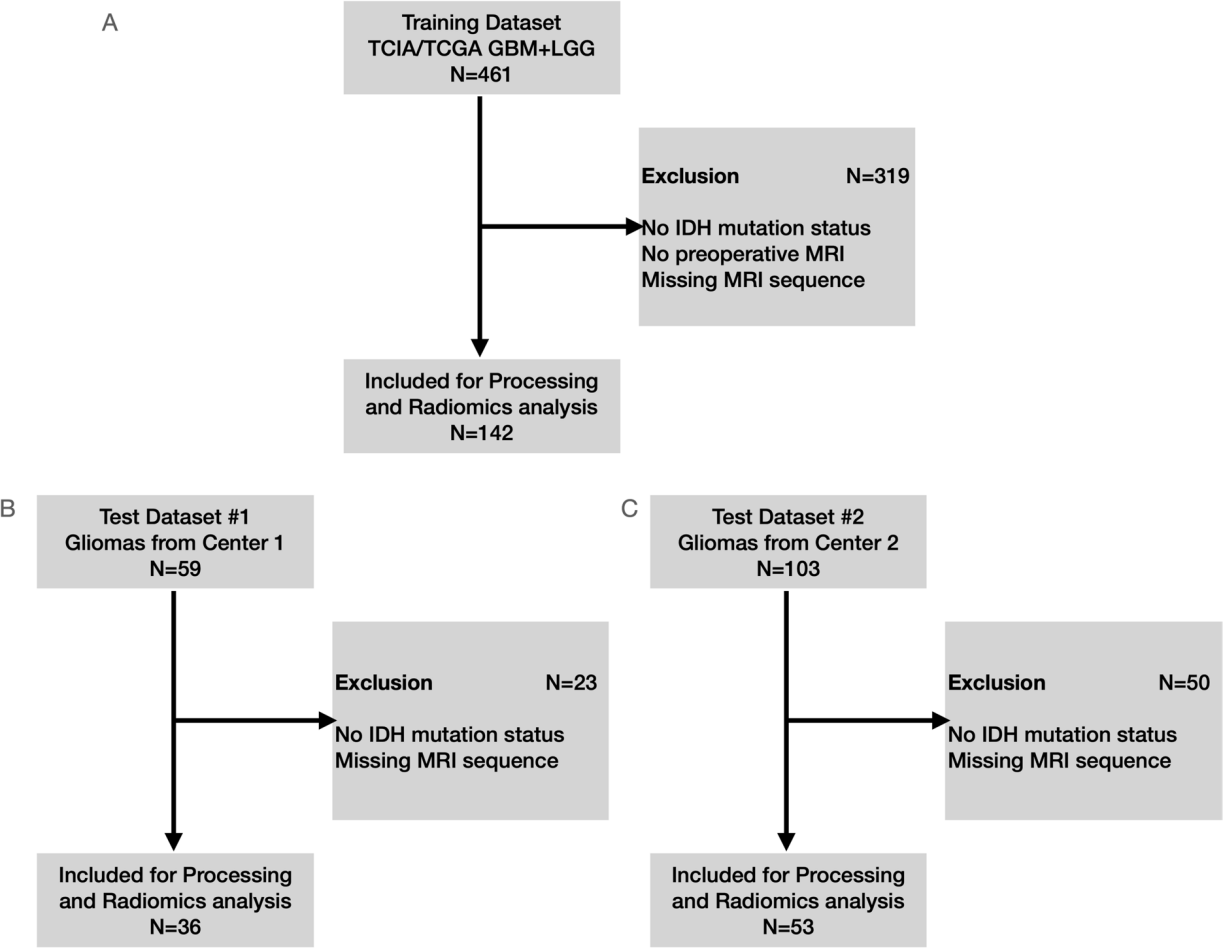
CONSORT diagram for **A** training, **B** test center #1, and **C** test center #2 patient selection

A total of 461 patients with glioma from The Cancer Imaging Archive were reviewed for selection of the training dataset, and 319 patients were excluded for several reasons, such as IDH mutation status unavailable, no pre-operative MRI sequences, or missing sequence. A total of 142 (32.4% were IDH wild-type) were included for processing, radiomic analysis, and subsequent model training. For the test dataset from center 1, 59 patients with glioma were reviewed, and 23 patients were excluded due to IDH mutation status unavailability or missing MRI sequence. On the test dataset from center 2, from the 103 patients with glioma reviewed, 50 patients were excluded for the same reasons. As a result, for the two external test datasets, 36 (63.9% were IDH wild-type) and 53 (75.5% were IDH wild-type) patients were included, respectively, from center 1 and center 2, for processing, radiomic analysis, and model evaluation. The CONSORT diagram depicting the selection process is shown in Fig. 1.

### Patient Characteristics

An overview of the patients in the training cohorts and the two independent test datasets is listed in Table 3.

**Table 3:** Description of the cohorts used in this study

		**Overall**	**Train cohort**	**Test cohort 1**	**Test cohort 2**	***p*-value**
***n***		231	142	36	53	
**Age, mean (SD)**		50.9 (15.4)	48.8 (14.3)	54.8 (16.3)	54.0 (16.8)	0.027
**Sex, *n* (%)**	**Female**	113 (48.9)	73 (51.4)	16 (44.4)	24 (45.3)	0.631
	**Male**	118 (51.1)	69 (48.6)	20 (55.6)	29 (54.7)	
**IDH, *n* (%)**	**Mutant**	122 (52.8)	96 (67.6)	13 (36.1)	13 (24.5)	< 0.001
	**Wild-type**	109 (47.2)	46 (32.4)	23 (63.9)	40 (75.5)	

In the training set, the IDH mutation status was wild-type for 46 patients (incidence of 32.6%), whereas, in the test datasets, 23 (63.9%) and 40 (75.5%) patients presented an IDH wild-type status in cohort 1 and cohort 2, respectively. The differences between the training and test sets were significant for the IDH mutation status (*p* < 0.001). Overall, the age distribution had a median value of 52.0 years, with the training and test cohorts 1 and 2 having median ages of 49.5, 54.5, and 60.0 years, where these differences were significant (*p* = 0.015). No significant differences were found in gender (females—overall, 48.9%; training, 51.4%; test cohort 1, 44.4%; test cohort 2, 45.3%).

### Radiomics Model Performance

**Fig. 2:**
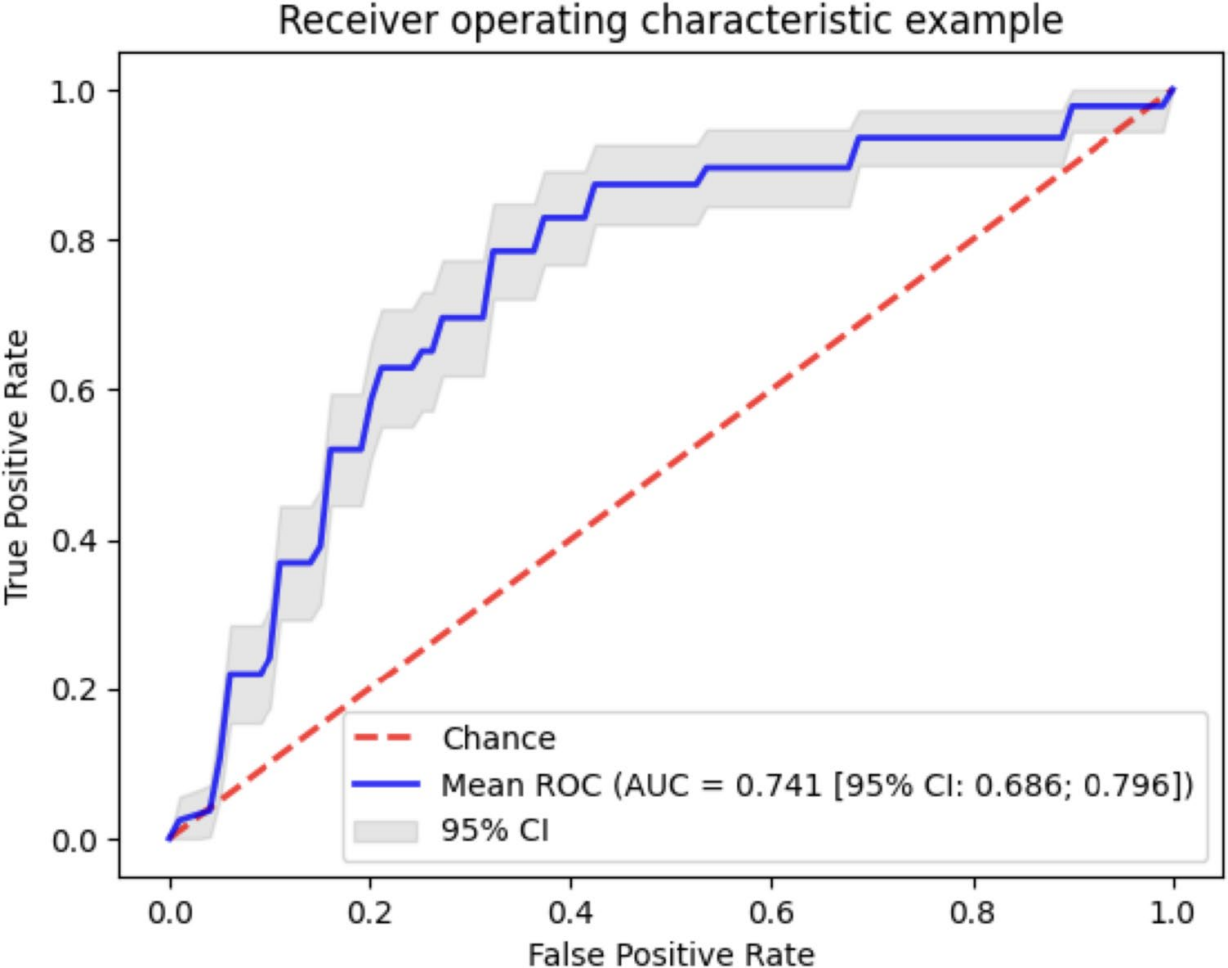
ROC curve and corresponding 95% confidence interval of the developed model. AUC, area under the curve; ROC, receiver operating characteristic

The regularization strength was optimized and the model was tuned using a fivefold cross-validation procedure. The cross-validation performance estimation yielded an AUC of 0.741 (95% CI, 0.686; 0.796), a sensitivity of 0.784 (95% CI, 0.721; 0.847), and a specificity of 0.657 (95% CI, 0.605; 0.709). The ROC curve of the training procedure is presented in Fig. 2.

**Fig. 3:**
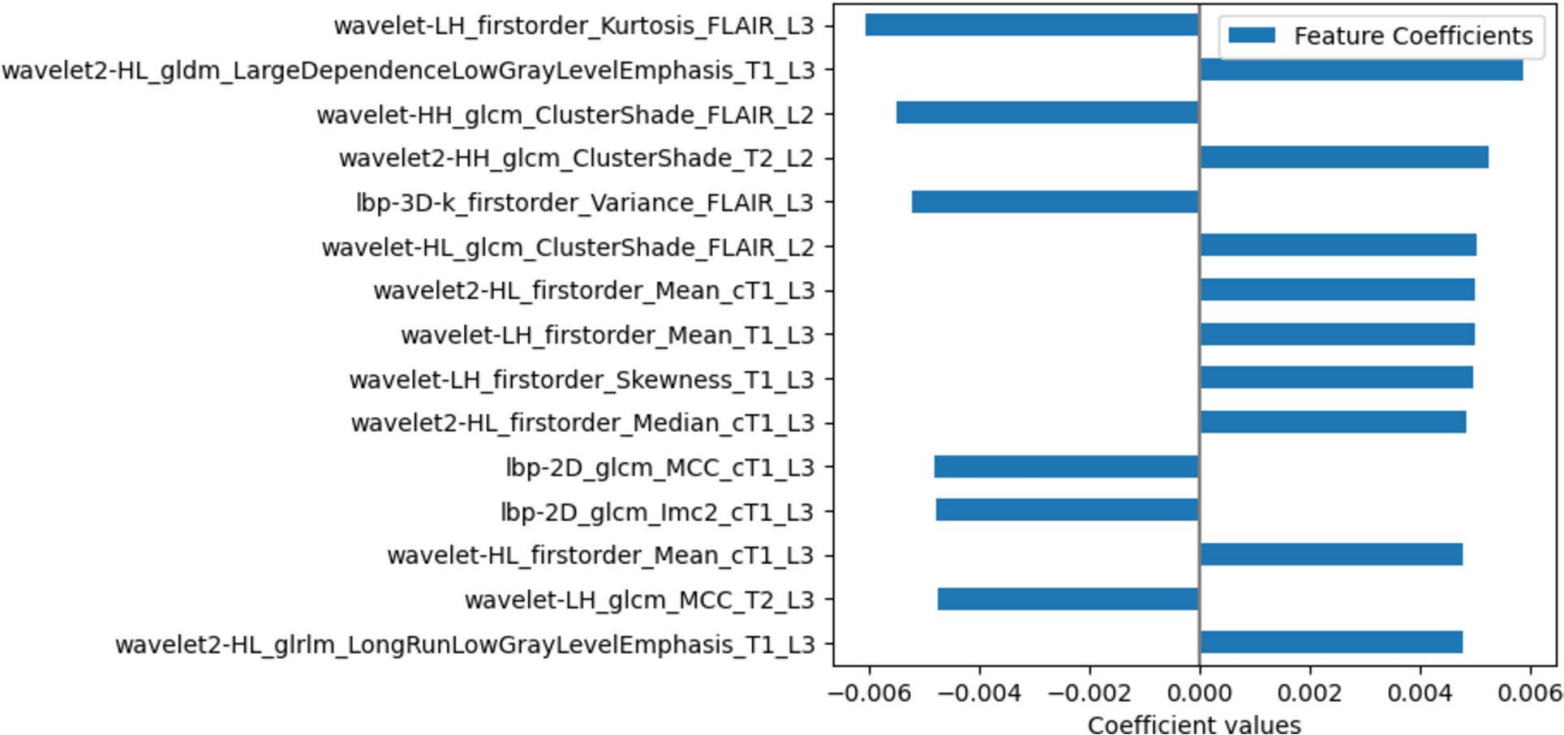
The subset of the 15 coefficients with the highest absolute value from the IDH mutation status predictive model

A subset of the 15 coefficients with the highest absolute value from the developed model is shown in Fig. 3. The full list of coefficients is provided at https://github.com/JoaoSantinha/End2EndAI_IDH_Prediction..

**Fig. 4:**
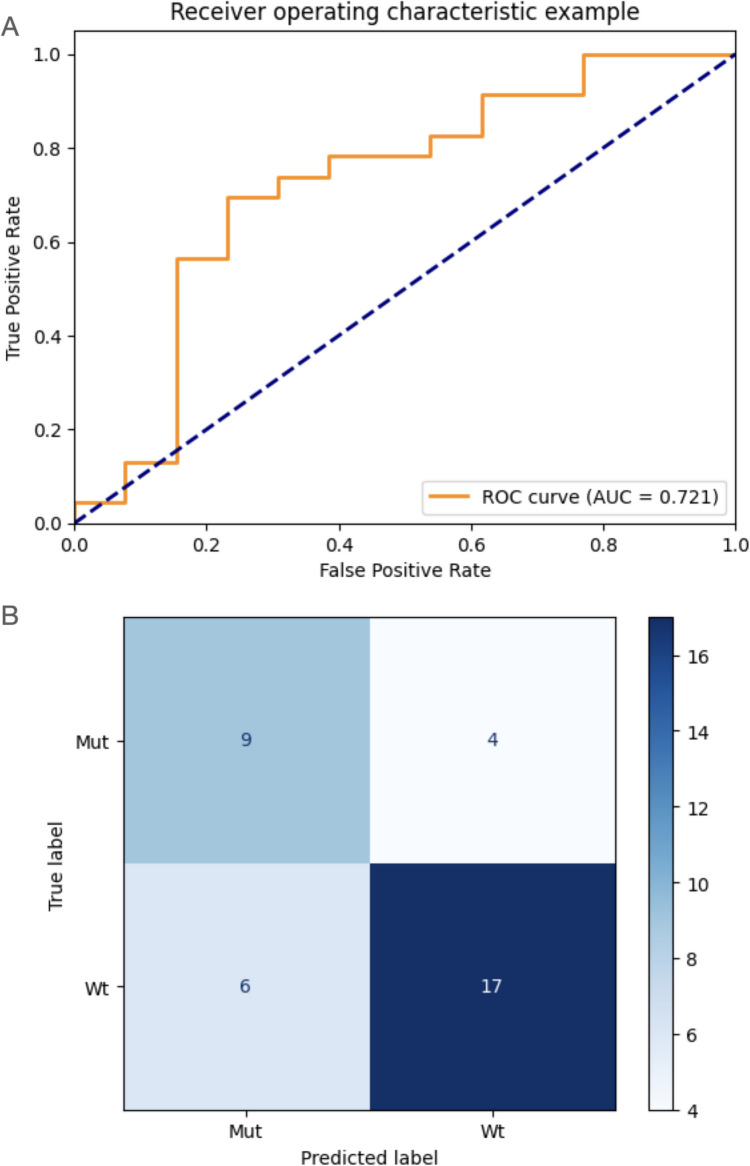
**A** ROC curve and **B** confusion matrix of the developed model on the test cohort 1. AUC, area under the curve; ROC, receiver operating characteristic; Mut, IDH-mutant; Wt, IDH wild-type

On the test cohort 1, the model achieved an AUC of 0.721 (95% CI, 0.574; 0.868) (ROC curve shown in Fig. 4A), a sensitivity of 0.739 (95% CI, 0.596; 0.882), and a specificity of 0.692 (95% CI, 0.541; 0.843). In Fig. 4B, the confusion matrix shows the number of correct and incorrect predictions. The calibration test yielded a *p*-value = 0.006 (calibration curve is shown in Supplementary Material Fig. S4A), indicating the poor model calibration on the external test cohort 1.

**Fig. 5:**
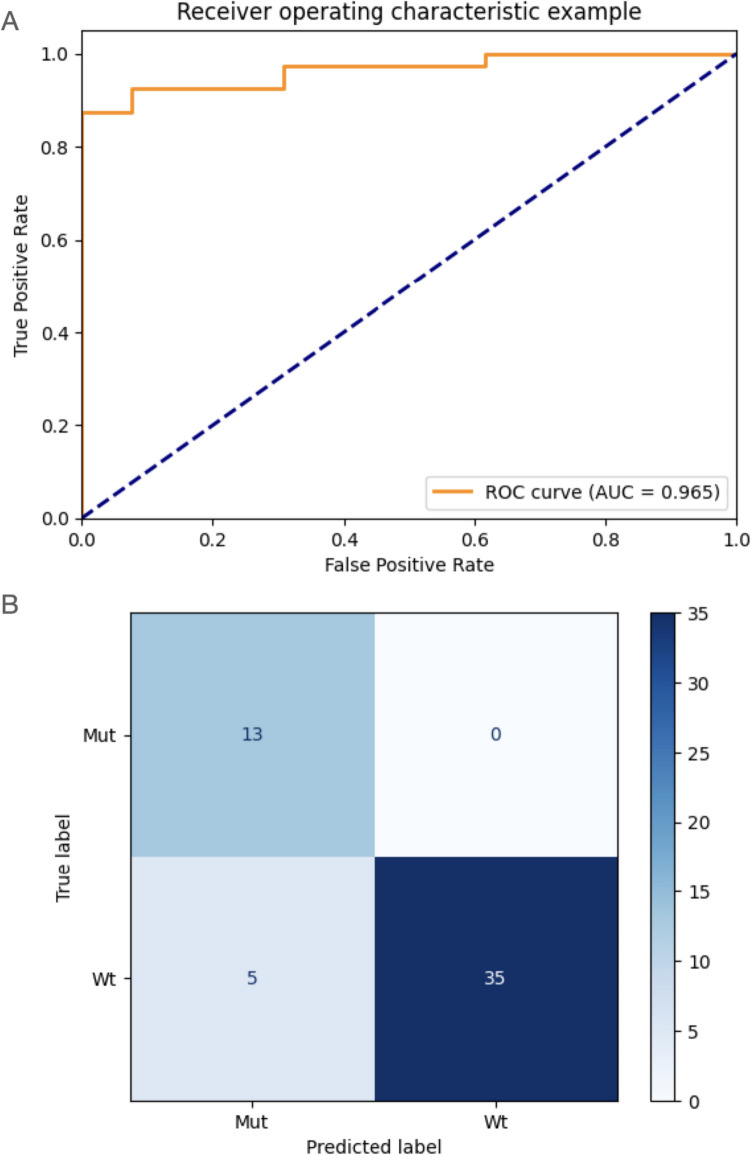
**A** ROC curve and **B** confusion matrix of the developed model on the test cohort 2. AUC, area under the curve; ROC, receiver operating characteristic; Mut, IDH-mutant; Wt, IDH wild-type

The developed predictive model showed on the test cohort 2 an AUC of 0.965 (95% CI, 0.837; 1.000) (ROC curve shown in Fig. 5A). At the operating threshold, the model showed a sensitivity of 0.875 (95% CI, 0.743; 1.000) and a specificity of 1.000 (95% CI, 0.874; 1.000). The corresponding confusion matrix showing the number of correct and incorrect predictions is shown in Fig. 5B. The calibration test on the external test cohort 2 showed a *p*-value > 0.001 (calibration curve is shown in Supplementary Material Fig. S4B), indicating poor calibration on this external test dataset.

The performance metrics obtained during training and on each of the test cohorts, as well as on both test cohorts unified, are shown in Table 4. Confusion matrices for each test cohort and WHO low- and high-grade gliomas according to WHO CNS classification prior to the 2021 revision are shown in Supplementary Material Fig. S5.

**Table 4:** Model performance metrics obtained during training and evaluation on the test cohorts. CV, cross-validation; CI, confidence interval; AUC, area under the curve

	**CV (95% CI)**	**Test center 1 (95% CI)**	**Test center 2 (95% CI)**	**Test center 1 + test center 2**
**AUC**	0.741 (0.686; 0.796)	0.716 (0.569; 0.863)	0.938 (0.809; 1.000)	0.836
**F1**	0.625 (0.573; 0.677)	0.773 (0.636; 0.910)	0.933 (0.803; 1.000)	0.874
**Accuracy**	0.698 (0.649; 0.747)	0.722 (0.576; 0.868)	0.906 (0.775; 1.000)	0.831
**Sensitivity**	0.784 (0.721; 0.847)	0.739 (0.596; 0.882)	0.875 (0.743; 1.000)	0.825
**Specificity**	0.657 (0.605; 0.709)	0.692 (0.541; 0.843)	1.000 (0.874; 1.000)	0.846

The developed predictive model with corresponding weights and instructions on how to use it is available in the following GitHub repository: https://github.com/JoaoSantinha/End2EndAI_IDH_Prediction..

### Age and Sex Biases Assessment

The assessment of potential age and sex biases was conducted, allowing a better characterization of the model performance across age groups and sex. As shown in Table 3, the proportion of females and males between the training and the test cohorts changed, with 51.4% of the training cases being females, and on the test cohorts of centers 1 and 2, 44.4% and 45.3%, respectively.

**Fig. 6:**
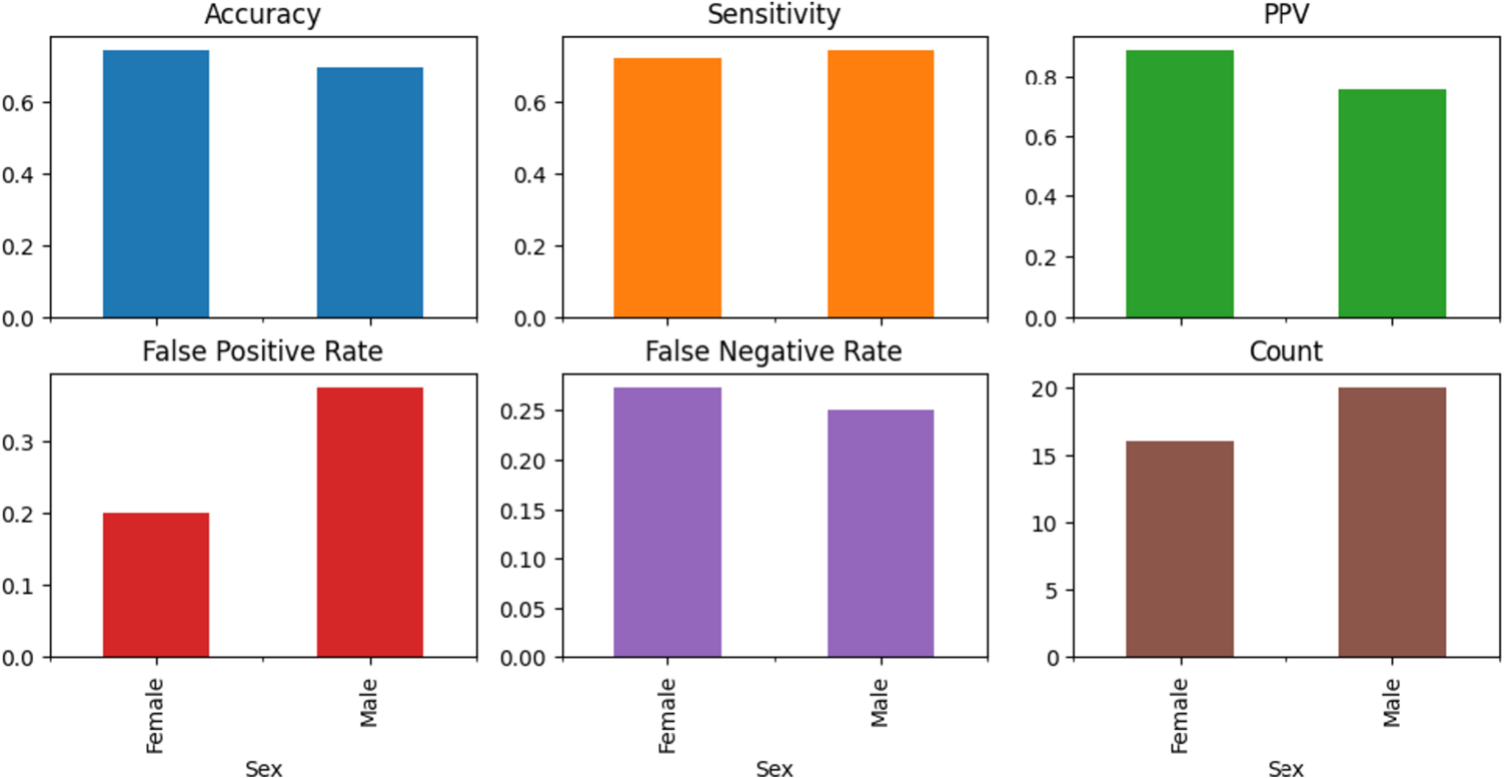
Model performance metrics on the test cohort from the center 1 group by sex. PPV, positive predictive value

Despite such differences, on the test cohort from center 1, the model sensitivity was similar for both females and males, with the model showing a slightly higher accuracy, positive predictive value, and false negative rate and a lower false positive rate in females, as shown in Fig. 6.

**Fig. 7:**
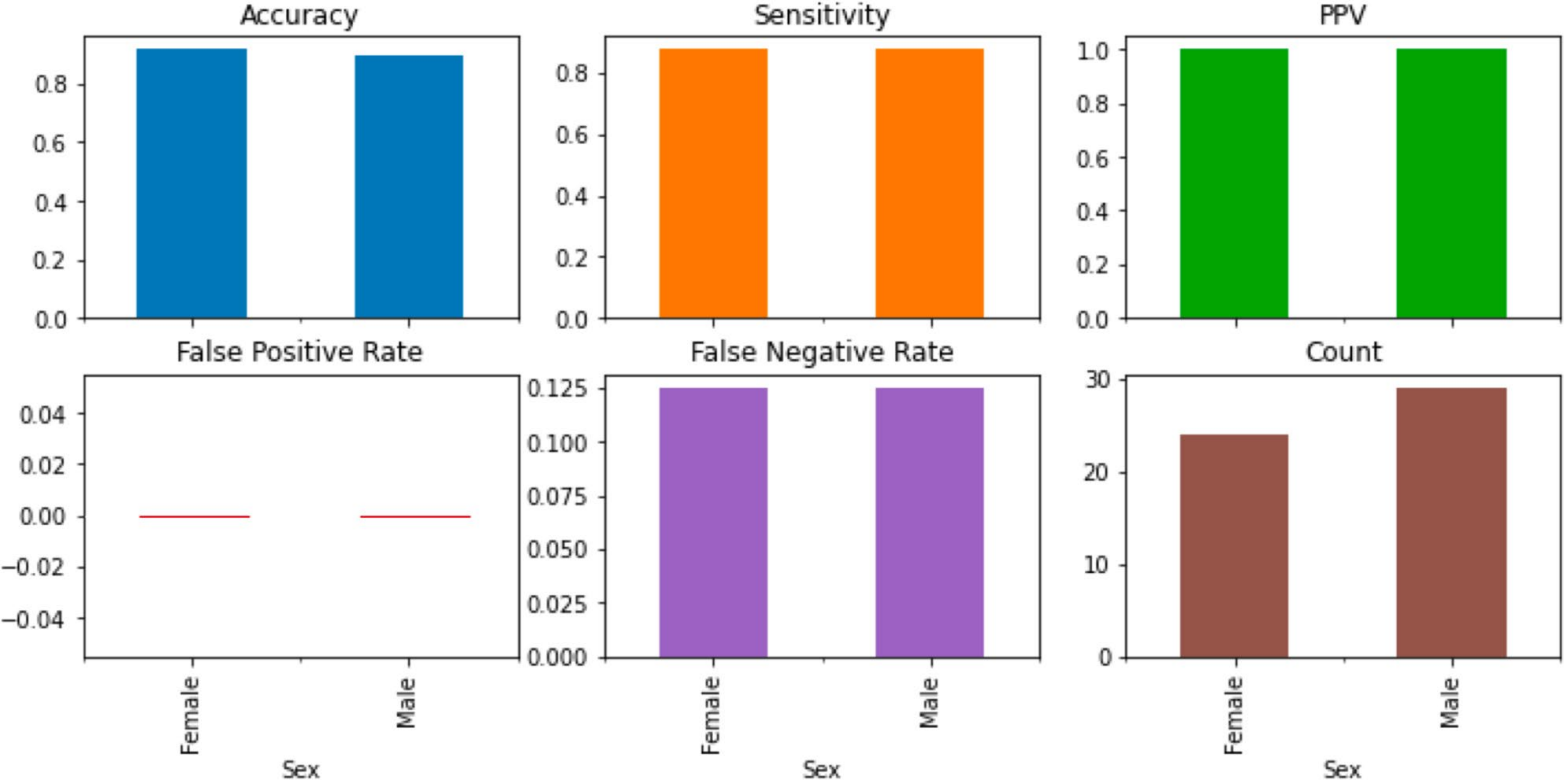
Model performance metrics on the test cohort from the center 2 group by sex. PPV, positive predictive value

On the test cohort from center 2, where the percentage of females was similar to the one from the test cohort from center 1, the model performance was equal for both females and males, achieving a 100% positive predictive value and a 0% false positive rate, as shown in Fig. 7.

**Fig. 8:**
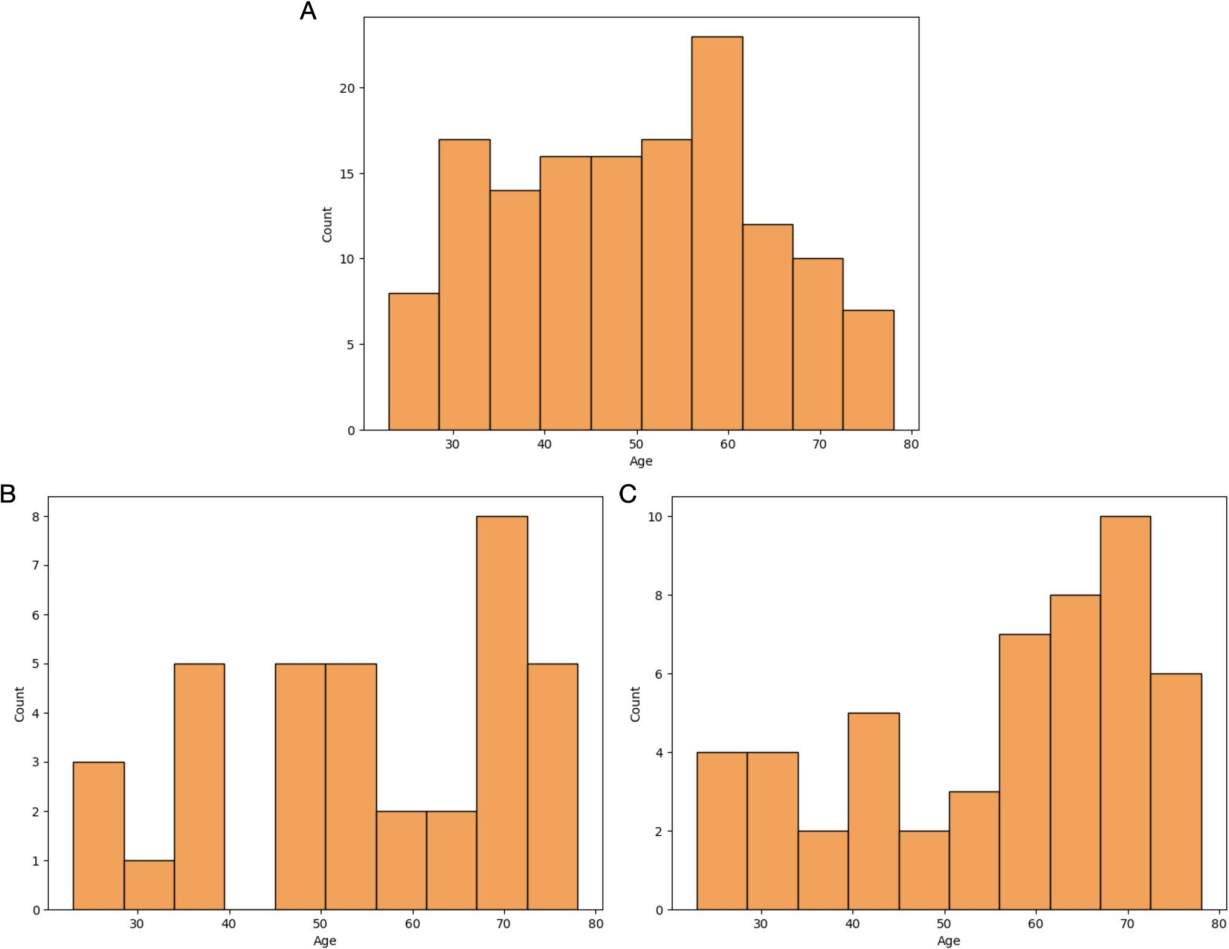
Age distributions of the training cohort (**A**) and test cohorts 1 (**B**) and 2 (**C**), respectively

Regarding the age distribution of the training and test cohorts which showed statistically significant differences (see Table 4), from Fig. 8, it is possible to observe that the age distribution of the test cohorts from centers 1 and 2 is skewed towards higher age groups compared to the one of the training cohort.

**Fig. 9:**
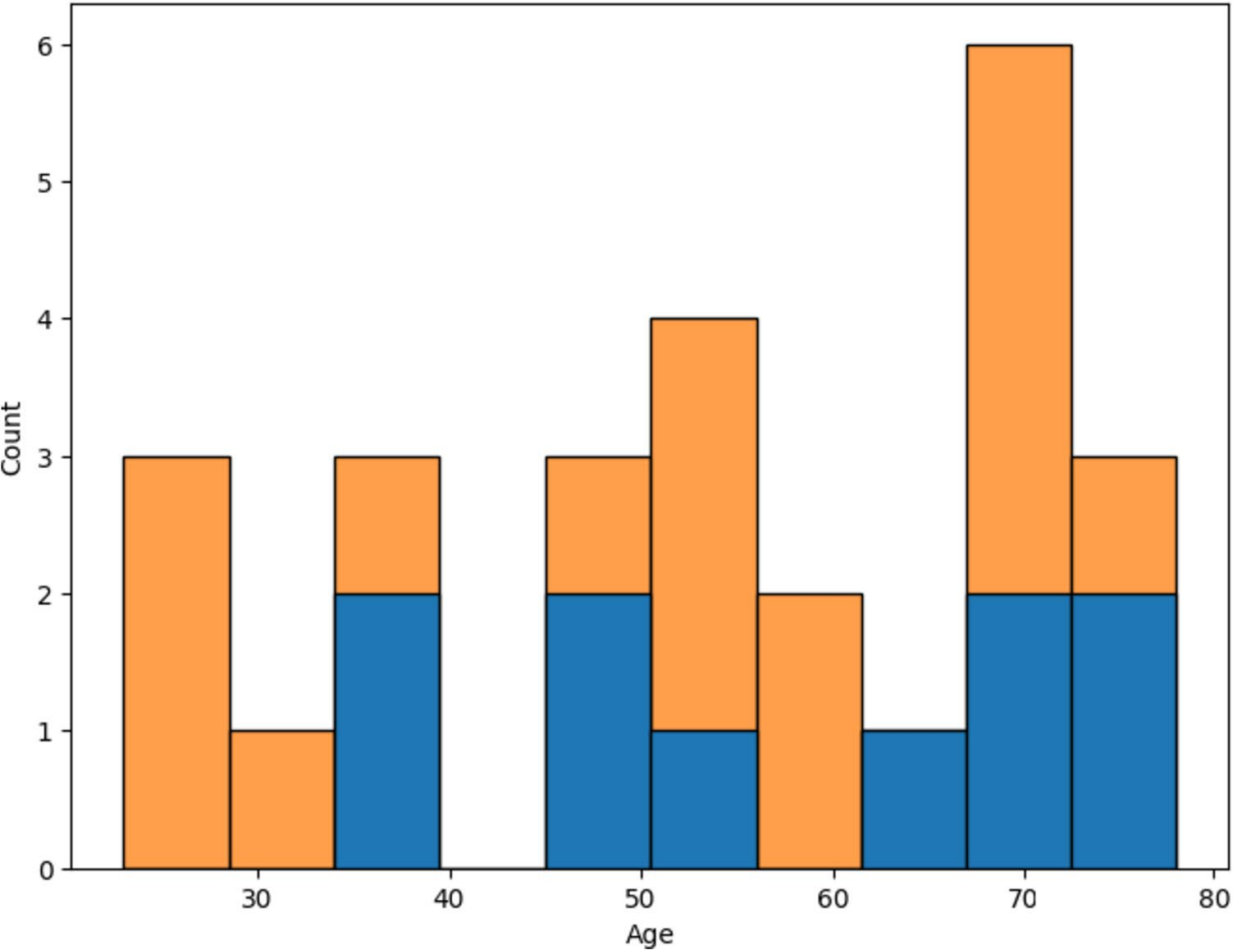
Model error distribution by age group (blue) and cohort age distribution for test center 1

**Fig. 10:**
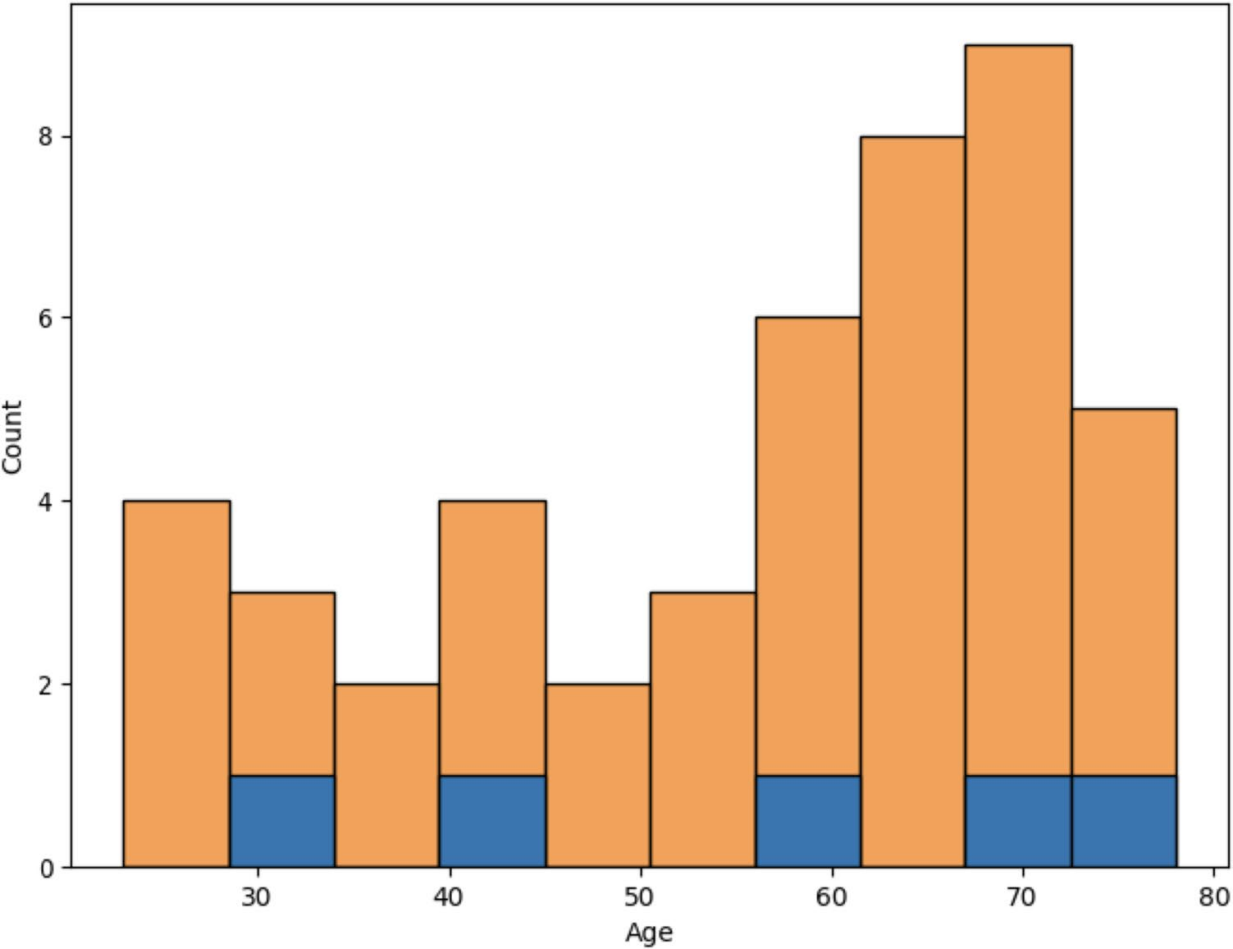
Model error distribution by age group (blue) and cohort age distribution for test center 2

The distribution of errors per age group for the test cohorts from centers 1 and 2 is shown in Figs. 9 and 10, respectively. In both test cohorts, no clear evidence of errors affecting particular age groups is observed, but the small sample size of each cohort does not allow the determination of model biases across age groups.

### TRIPOD Statement

The TRIPOD adherence was determined, and a score of 94% was obtained. The detailed adherence form for this development and validation study is available in Supplementary Material Table S2.

## Discussion

Following the recent WHO CNS reclassifications of glioma (2016 and 2021 updates), the IDH gene mutation status has become a significant stratification and prognostic factor for patients with gliomas, regardless of the histologic grade [2–4]. Even though the standard glioma treatment is maximum tumor resection, the determination of IDH mutation status can help post-surgery treatment planning and provide such information in areas of the world where genomic analysis may not always be available.

In this multicenter study, we developed and evaluated a radiomics model to predict IDH mutation status using radiomic features extracted from standard-of-care pre-operative MRIs. The radiomic features are extracted from automatically defined regions of interest comprising the contrast-enhancing, necrotic/non-contrast-enhancing, and edema regions using a pre-existing deep learning segmentation model. The radiomics model was developed using the TCIA/TCGA glioma dataset, which comprises the previous definition of glioblastomas (TCGA-GBM) and low-grade gliomas (TCGA-LGG). The model assessment was performed on two independent European centers, presenting a significantly higher prevalence of patients with IDH wild-type than in the training cohort. In addition, the adherence to TRIPOD of the model development and validation was determined, having obtained a high score.

Observing Fig. 3, it is possible to understand that features from the edema (L3) and non-enhancing/necrotic regions (L2) with no feature from the enhancing region (L1) were present and that cT1w was the sequence with features having higher coefficients followed by FLAIR, T1w, and T2w. Interestingly, all features were obtained from filtered images, and no shape features were present.

The performance of the developed model on the test center 1 cohort showed an AUC, accuracy, sensitivity, and specificity within the training cross-validation 95% CIs, despite lower AUC and sensitivity and higher specificity and accuracy. Regarding the F1-score, the model yielded a higher score than the training cross-validation 95% CI, which can be justified by the considerably lower number of false negatives despite the slightly lower sensitivity. Interestingly, the model performance on the test center 2 cohort was considerably higher than the results obtained on the cross-validation during training across all performance metrics. A possible reason for such results may lay in a similar quality and contrast of the images obtained in the test cohort from center 2 with some of the images from hospitals constituting the training cohort, which may have resulted in more accurate predictions, as the distribution of intensities between images of the test cohort from center 2 was close to some of the samples in the training cohort (from the comparison between Supplementary Material Table S2 and Table 2 of [21]). Despite the poor calibration on both test cohorts, which shows that the model would need to be calibrated to each external institution to use it as a risk model, the discriminative model performance on test cohorts 1 and 2 yielded results similar or better than the cross-validation performance. Furthermore, in the development of the model using a cohort of patients from a different continent, the analysis of the model’s fairness in terms of age and sex did not show, as desired, a clear bias in any of these variables.

Despite the large number of studies investigating the use of radiomics to predict IDH mutation status in grade II and III gliomas [31–34], a much smaller number of studies have developed and assessed IDH mutation status prediction models with cohorts of patients based on the most recent WHO CNS glioma reclassifications from 2016 and 2021 [13–15, 25, 26]. In [25] and [26], the authors used the TCIA/TCGA glioma dataset and iteratively left one institution as the external test set and the remaining as the training dataset. In the first study, performances of the proposed model achieved sensitivities/specificities on the test datasets of 0.67/0.75, 0.00/0.82, 0.86/0.72, and 0.53/0.71 on each iteration, respectively. The sample sizes of these datasets were relatively small, comprising 7 (3 wild-type cases), 12 (3 wild-type cases), 36 (7 wild-type cases), and 22 cases (15 wild-type cases). Similarly, in the second study but with smaller sample sizes (number of patients (number of wild-type cases)—35 (7); 11 (5); 5 (1); 12 (1)), the proposed IDH mutation status prediction model achieved sensitivities/specificities on the test datasets of 0.571/0.821, 0.800/0.667, 1.000/1.000, and 1.000/0.818. Despite yielding better performances in some scenarios than the current proposed model on the test cohorts, our study assessed the developed model using two independent test datasets with larger sample sizes and an end-to-end AI–based approach, where human intervention is not required. In the study by Choi and colleagues [13], the deep learning model yielded accuracies on the two test cohorts of 0.841 and 0.735, while the radiomics model showed accuracies of 0.794 and 0.754. The absence of sensitivity and specificity values for each model on test cohorts requires the analysis of the patient characteristics for a fairer comparison. The IDH mutation status distributions of their first test dataset match closer to our second test dataset, while their second test dataset to our first test dataset. Based on this assumption, the model proposed in this study shows high accuracy for our test dataset 2 and lower for our test dataset 1 when compared with their two approaches, with the limitation that our test datasets have smaller sample sizes. The model developed by Manikis and colleagues [14] used radiomic features extracted from DSC-MRI, achieving an accuracy of 0.71, an F1-score of 0.47, an AUC of 0.67, a sensitivity of 0.60, and a specificity of 0.74 on the external validation comprising two independent cohorts. Finally, the model developed by Sudre et al. [15] also using radiomic features extracted from DSC-MRI yielded a sensitivity of 0.77 and a specificity of 0.65 but was assessed only through stratified twofold cross-validation with 250 repetitions. Despite this model evaluation difference, the use of standard-of-care MRI images, and the end-to-end AI–based analysis, the weighted averages of test sensitivities and specificities of the model proposed (presented in Table 4) were 0.825 and 0.846, showing a better performance. Additionally, these studies shared the absence of fairness evaluation, which was performed in our study to assess potential age and sex biases of the proposed model.

Despite evaluating the model using two independent cohorts of patients, these datasets were of small sample size, representing a limitation of the current study. Furthermore, despite the racial information being available for the training dataset, such information was not recorded and available for the test cohorts, preventing the assessment of potential racial biases of the developed model. Another limitation tied to the availability of only two independent patient cohorts was the restriction it posed on training and evaluating multiple classifiers on external test cohorts before proceeding to the validation. This, in turn, hindered the subsequent process of selecting the most optimal classifier and conducting the model validation on unseen independent cohorts.

In future work, more advanced tumor habitat definitions could capture better tumoral heterogeneity, potentially resulting in better model performance and robustness. The inclusion of more independent cohorts could allow the assessment of other classifiers (e.g., random forest, gradient boosting, among others) at the testing phase and still perform the validation on unseen independent cohorts. Additionally, radiomic feature maps would potentially be helpful to guide stereotactic brain biopsies towards more aggressive tumoral regions, which could lead to a more accurate diagnosis and treatment selection.

## Conclusion

A radiomics model to predict IDH mutation status in patients with glioma was developed and assessed using multicentric data. The model showed good generalizability and robustness when applied to the external test cohorts with no evidence of sex and age biases.

### Supplementary Information

Below is the link to the electronic supplementary material.

Supplementary file1 (DOCX 2534 KB)

## Data Availability

The training data presented in this study are openly available in https://www.cancerimagingarchive.net at https://doi.org/10.7937/K9/TCIA.2016.L4LTD3TK and https://doi.org/10.7937/K9/TCIA.2016.RNYFUYE9. The test data presented in this study are available on request from the corresponding author. The test data are not publicly available due to privacy restrictions. The developed model is available in https://github.com/JoaoSantinha/End2EndAI_IDH_Prediction.
